# Effects of Myofascial Induction Therapy on Ankle Range of Motion and Pressure Pain Threshold in Trigger Points of the Gastrocnemius—A Clinical Trial

**DOI:** 10.3390/biomedicines11092590

**Published:** 2023-09-21

**Authors:** Eva María Martínez-Jiménez, Raquel Jiménez-Fernández, Inmaculada Corral-Liria, David Rodríguez-Sanz, César Calvo-Lobo, Daniel López-López, Eduardo Pérez-Boal, Bibiana Trevissón-Redondo, Jessica Grande-del-Arco

**Affiliations:** 1 Nursing Department, Faculty of Nursing, Physiotherapy and Podiatry, Universidad Complutense de Madrid, 28040 Madrid, Spain; evamam03@ucm.es (E.M.M.-J.); davidrodriguezsanz@ucm.es (D.R.-S.); cescalvo@ucm.es (C.C.-L.); jessgran@ucm.es (J.G.-d.-A.); 2 Department of Nursing and Stomatology, Faculty of Health Sciences, King Juan Carlos University, Alcorcon Campus, 28922 Madrid, Spain; inmaculada.corral.liria@urjc.es; 3 Research, Health, and Podiatry Group, Department of Health Sciences, Faculty of Nursing and Podiatry, Industrial Campus of Ferrol, Universidade da Coruña, 15403 Ferrol, Spain; daniellopez@udc.es; 4 Department of Nursing and Physiotherapy, Faculty of Health Sciences, Universidad de León, 24007 León, Spain; epereb@unileon.es

**Keywords:** range of motion, foot, trigger point, myofascial pain syndrome, pain pressure threshold

## Abstract

Background: The myofascial induction technique (MIT) has been shown to increase shoulder range of motion (ROM) in breast cancer survivors and decrease pain pressure threshold over the radial nerve in patients with epicondylalgia. To the authors’ best knowledge, no study on trigger points and MIT has been published to date. The effect on ROM of latent trigger points is also unknown. Methods: A total of 20 twins with one latent trigger point of the gastrocnemius muscle were evaluated pre- and post-MIT in the calf. We measured static footprint variables in a pre–post study. Results: We found differences in PPT (*p* = 0.001) and no differences in ROM with knee flexed (*p* = 0.420) or stretched (*p* = 0.069). Conclusions: After Calf MIT, latent myofascial trigger points improve PPT but no change in ankle dorsiflexion with knee bent or knee flexed were found in non-restriction healthy subjects.

## 1. Introduction

The most common musculoskeletal condition is the so-called myofascial pain syndrome (MPS). Its prevalence is estimated at 85% of the general population. MPS is defined as a set of sensory, motor, and/or autonomic signs and symptoms in a tight band of skeletal muscle. These signs and symptoms are caused by hyperirritable nodules, which are called myofascial trigger points (MTPs) [1].

A latent myofascial trigger point (MTP) is defined as a focus of hyperirritability in a taut muscle band and is clinically associated with local twitch responses and tenderness and/or referred pain upon manual examination [2,3,4]. Considering the patient’s level of pain recognition in the spine, MTPs may be active or latent [2,3,4]. Both types generate clinical symptoms consisting of decreased joint range and increased fatigue, but only the latent trigger point does not present pain unless it is mechanically stimulated [5]. Active MTPs generate spontaneous and recognized pain [4]. Second, latent MTPs may produce local or referred pain after stimulation [2]. Latent MTPs are as prevalent in patients with different spinal conditions as in healthy subjects [3]. Nevertheless, both MTPs show differences in electrophysiological activity levels and biochemical milieu [6]. Mechano-sensitivity, as determined by means of the pressure pain threshold (PPT), was shown to differ between active MTPs, latent MTPs, and normal control sites [7]. Latent trigger points can be objectively verified in various ways, including intramuscular electromyography, since spontaneous electrical activity is observed as in active trigger points. Using ultrasound, it is possible to observe a hypoechoic image with a reduced vibration amplitude in such cases [8,9]. Latent MTPs can be easily activated by changing their pain sensitivity from stimulus-induced to spontaneous thus generating an active MTP [2,8]. Latent MTPs contribute to several musculoskeletal problems: (1) local tenderness without referred pain and (2) local tenderness with referred pain upon mechanical stimulation with subsequent development of cramps, decreased joint range, muscle weakness, fatigue, deficits in fine muscle control, and alternating muscle activation patterns [9]. Assessment of latent MTPs can be done by means of thumb palpation, pressure pain threshold (PPT) determination, intramuscular needle electromyography, surface electromyography, infrared thermography, and laser Doppler flowmetry [9].

Manual mechanical stimulation or needle insertion of latent trigger points with increased sensitivity induces centrally mediated referred pain; therefore, it is not surprising that latent MTPs have the potential to induce sensitization. Apart from referred pain, which occurs a few seconds after mechanical stimulation of latent MTPs, it is known that after such mechanical stimulation, the mechanical pain threshold decreases within a few minutes [10]. The pain at an active trigger point is constant and spontaneous, which seems to indicate that it forms new central nervous connections, and that in the case of latent MTPs, these connections in the central nervous system are not as effective as those normally present [11]. The system is now in a sensitized state as found in acute cases and chronic pain conditions in which MTP sensitivity would increase to an even higher level [12]. The clinical importance of these findings is that treatment of latent MTP in patients with chronic diseases, produce a decrease in mechanical pain, hyperalgesia, and allodynia but also prevent the transition to active MTPs. In addition to motor dysfunctions, an alteration in sensitivity occurs [2].

MTPs in the calf musculature are common, especially in runners for whom dry needling has been shown to be effective both in treating pain and producing improvements in joint range in ankle dorsiflexion [13]. Latent trigger areas are strongly related to many other lower limb disorders and dysfunction, such as patellofemoral pain, medial or lateral meniscal injuries leading to surgical procedures, and lower limb joint osteoarthritis affecting normal ranges of motion [14]. On the basis of the best available evidence, dry needling seems to be effective in facilitating a reduction in pain that is related to lower extremity MTPs [14].

Myofascial induction therapy (MIT) is a manual therapy used in physiotherapy and focuses on restoring altered fascial tissue. In a healthy body, the fascial system maintains elasticity and coordination of movements. However, injuries and their after-effects, such as scars, may reduce this system’s role, thus leading to dysfunctional movements [15].

The MIT technique has demonstrated different effects in research. After eight sessions of MIT application to a scar fold, functional improvement of the skin as determined by the Schober test was observed [15]. After an MIT session in patients with low back pain, statistical analyses revealed a significant increase in the contraction speed of the lumbar erector spinae in subjects with low back pain [16]. Range-of-motion (ROM) of the shoulder was measured in breast cancer patients after applying MIT, and it was found that a single MIT session produced a decrease in pain and an increase in shoulder ROM more than an electrotherapy session [17].

Mechano-sensitivity and joint range versus placebo after a single session of MIT were also measured in patients who had survived breast cancer. A single MIT session produced a decrease in pain intensity and improvement in neck–shoulder ROM to a greater degree than did the control electrotherapy session. The MIT was performed based on the PPT records of the affected and unaffected lower limbs and produced an improvement in mechano-sensitive properties of the radial nerve of the affected upper limb thus improving its ROM. No differences were found when comparing the other nerves [16].

Existing research in healthy subjects in a cohort study indicates that the application of MIT to the cervical area produces an improvement in joint range, specifically cervical flexion, extension, and left lateral flexion but not rotation motion. No changes, however, in PPT in either the C5–C6 zygapophyseal joint (local point) or tibialis anterior muscle (distant point) were found [18]. On the other hand, MIT application to the masseter and temporalis muscles in healthy subjects without pain produced no significant differences in maximum vertical mouth opening, mechanical sensitivity of the masticatory muscles, and head posture when compared with a placebo group [19].

Current evidence suggests that MIT is considered effective for treating chronic musculoskeletal pain in the upper limbs and thorax of female breast cancer survivors [20]. In chronic low back pain, MIT is shown to produce improvements in function, a decrease in pain, and improvements in quality of life and spinal flexion mobility in this region [21].

When this technique was applied to the lower limb plantar fascia, it was found to facilitate improvements in plantar pressures and balance [22]; however, no further studies have been conducted in this region.

Dommerholt believes that fascia can affect the pathophysiology of MTP, and other authors even propose to treat MTP with fascia-targeted treatments for this reason. However, with regard to evaluating improved range of motion, current research has only assessed self-myofascial release with a foam roller [23].

The gastrocnemius muscle has two important functions: (1) dynamic, together with the soleus muscle these muscles are the main plantar flexors of the ankle and (2) proprioceptive, which it performs in conjunction with the pretibial muscles, such as the tibialis anterior [24].

However, no study exists that has evaluated the effects of MIT application on lower limb or gastrocnemius on PPT trigger points or ankle joint range.

The purpose of this study was to test the effects on joint range in dorsal ankle flexion and the effect on the pain pressure threshold of latent trigger points of the calf after application of myofascial induction technique. We hypothesized that application of the MIT technique to the calf will produce improvements in joint range in dorsiflexion of the ankle and a reduction in PPT on the trigger point.

## 2. Materials and Methods

### 2.1. Sample Size Calculation

We calculated the sample size using G*Power 3.1.9.7 software (G*Power ©; University of Düsseldorf, Düsseldorf, Germany). The aim of this calculation was to find the sample size necessary to validate the objective of the study, namely, to observe the differences before and after MIT application to the calf in PPT and ankle ROM variables. We used a study with a similar methodology where the aim was to investigate the effects of 5 min of sustained stretching on ankle ROM. The authors found a 1.40 ± 3.30 degree increase after stretching. A total of 18 subjects were required for the study [25]. Thus, for the calculation of the sample analysis, we defined the type of study as a test of differences of related samples, namely, the same group pre- and post-intervention with a 2-tailed hypothesis, an effect size of 0.70, a probability of error of 0.05, and power of 0.80, a sample size of 19 subjects was obtained. As we evaluated two calves from each subject and performed MIT on each calf, a total of 10 subjects were recruited with the intention of obtaining data from 20 calves. The study was registered at clinicaltrials.gov as NCT05711745.

### 2.2. Subjects

A total of 10 subjects (20 calves) aged 19–26 years were recruited, three of them male and seven females. Socio-demographic data of all subjects and separate by gender could be seen in Table 1. Inclusion criteria consisted of subjects with trigger point 1 of the gastrocnemius muscle [26] in both lower limbs to fulfil the objective of observing the effects of MIT on PPT and ankle ROM.

Participation selection and inclusion criteria consisted of two parameters: (1) Untrained healthy individuals; the participants did not perform sports activities and were not engaged in strength or stretching exercises [27,28] and (2) age greater than 18 years and younger than 40 years.

Exclusion criteria for the study consisted of several parameters: (1) Diagnosis of lower limb injury, including any tendinopathy, bursitis, ligamentous involvement, and/or fasciitis [25]; (2) history of lower limb surgery or history of lower-extremity injury with residual symptoms (pain or feeling of sensations) within the last year [26]; (3) participants could not have undergone ankle stretching or any other treatment [21]; (4) diabetes due to possible alteration of arterial distal circulation [28]; (5) foot deformity, cavus, and flat feet; (6) two deformities, such as hammer toes and hallux valgus, (7) plantar corns and calluses, and/or (7) lower limb dysfunction or chronic injury [29].

All subjects signed an informed consent form before participating in the study. In relation to the ethics committee, the principles of the Helsinki declaration were followed. The ethics committee of the Rey Juan Carlos University approved the study with number 2911202021420.

### 2.3. Myofascial Induction Therapy

**Figure 1:**
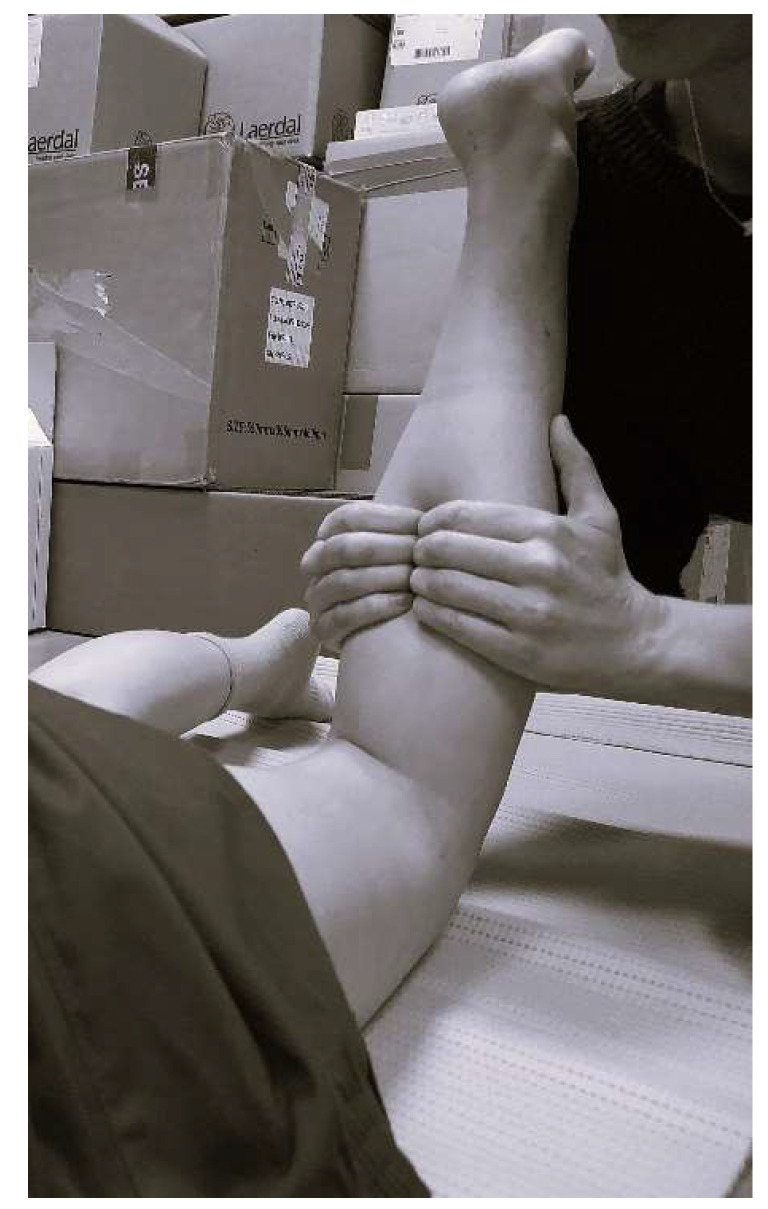
Position of Myofascial Induction Therapy of deep calf fascia.

The MIT intervention was performed based on the procedure described by Pilat to release the superficial fascia from the fascia. In this procedure, in the area behind the tibia, 3 longitudinal slides are performed from the medial malleolus to the knee and later the maneuver for the deep fascia is performed, where the therapist’s fingers are flexed and the fingertips are used to apply pressure maintained in the direction of the tibia (Figure 1). Both maneuvers were performed as described by Pilat and were performed by a clinician with more than nine years of experience (EMMJ). The second maneuver was held for a minimum of 5 min. Both legs received the same treatment while the subject was in prone position [30].

### 2.4. Measurement

#### 2.4.1. ROM Measurements

The same parameters were measured pre- and post-intervention.

**Figure 2:**
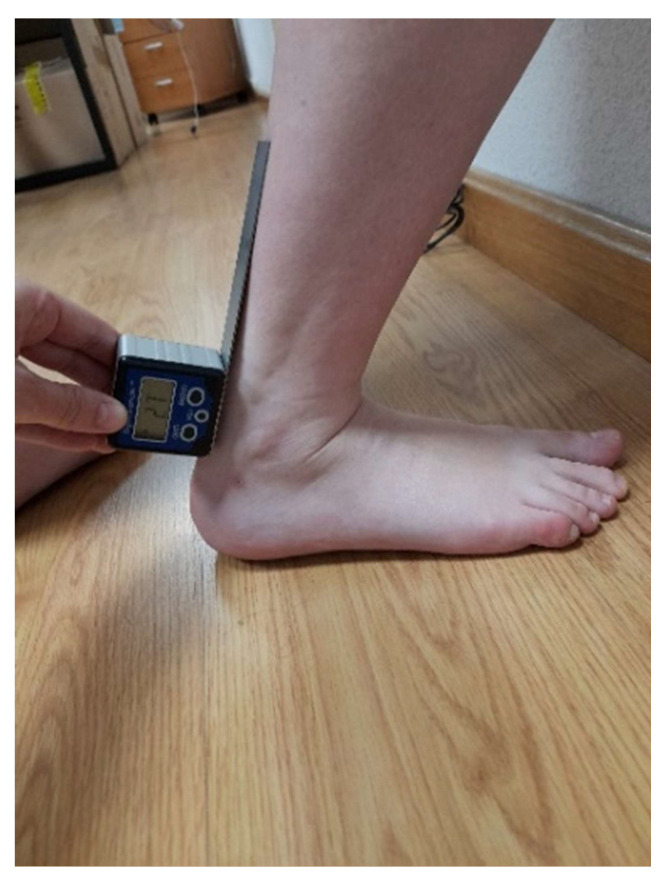
Position of joint range measurement position with bent knee.

The weight-bearing lounge test (WBLT) position measured with an inclinometer was used to measure the flexed knee. Weight-bearing stride was performed standing with the bent knee aligned with the second toe and the toe 10 cm from the wall. The heel always maintained contact with the ground. The foot then moved 1 cm away from the wall without separating the heel from the ground or taking the knee off the wall. If the knee moved away from the wall or the heel was lifted, the measurement was taken at this place at a maximum distance but in contact with the wall. The WBLT has demonstrated strong evidence that inter-clinician reliability (ICC = 0.80–0.99) as well as intra-clinician reliability (ICC = 0.65–0.99) considered a good reliability. Additionally, average MDC scores of 4.6° for inter-clinician and 4.7° for intra-clinician were found in the last metanalysis [31]. The stable arm of the goniometer was aligned with the ground and the mobile arm was aligned with the diaphysis of the fibula [32] as shown in Figure 2. We used a Digital Inclinometer Electronic Goniometers 4*90 Degree Magnetic Base Digital Protractor Angle (Neoteck®, Falcon e-fulfillment GmbH, Pfungstadt, Germany) with 1° increments that was previously demonstrated to be reliable in weight-bearing lunge dorsiflexion ROM measurement averages in research studies [33].

**Figure 3:**
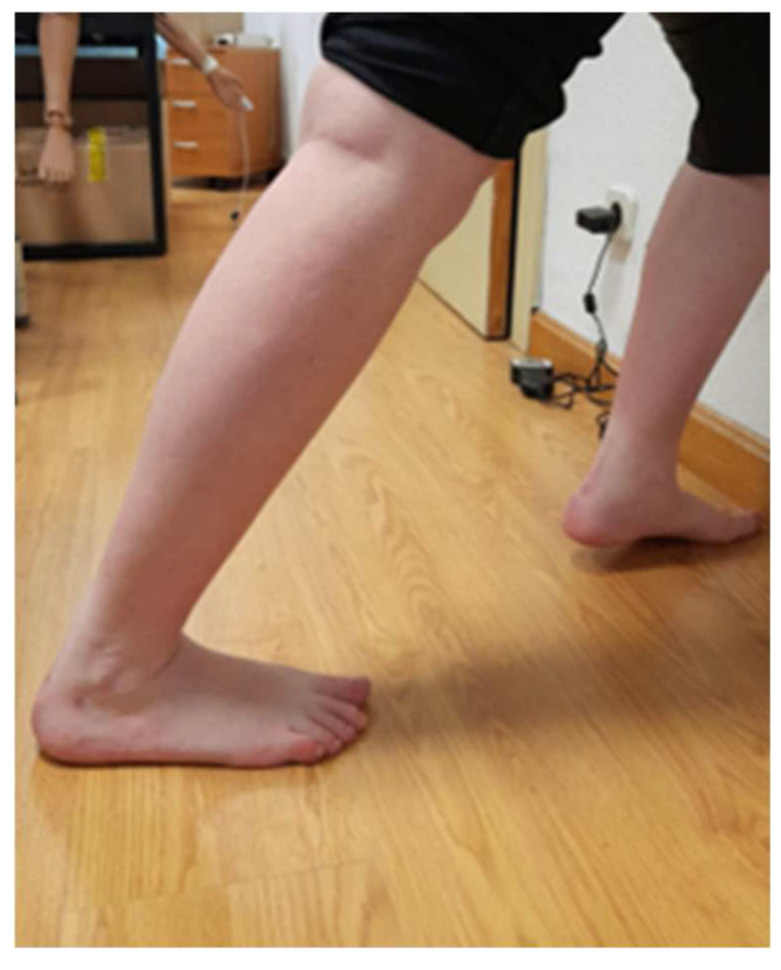
Position of weightbearing technique for the measurement of ankle joint dorsiflexion with the knee extended.

Subsequently, a weightbearing technique for the measurement of ankle joint dorsiflexion with the knee extended was used because of its proven reliability, Intra-rater reliability of the experienced raters was high for digital inclinometer an average ICC = 0.88, average 95% limits of agreement = −6.6 degrees to 4.8 degrees [34]. In this position, the subject faces the wall and supports his hands. The subject should try to keep the foot further and further back without lifting the heel or bending the knee. As soon as the knee is lifted or bent, the subject will bring the foot closer to the wall, as shown in Figure 3.

Measurements and intervention were obtained and conducted for both feet. A total of three measurements under each condition were recorded for each foot.

#### 2.4.2. Pain Pressure Threshold Measurements

Pain is specific to each person and can be reliably measured with an FDX 100 algometer from Wagner Instruments®, Greenwich, USA (www.wagnerinstruments.com), a digital algometer with capacity of graduation (500 × 0.5 N) that is reliable for superficial trigger point research [35]. Three recordings were obtained from the latent trigger point 1 of the inner calf as described by Travell and Simons once this point was located by the clinician with more than 10 years of experience in its localization and diagnosis. Three recordings were obtained pre- and post-intervention for both lower limbs.

### 2.5. Variables

The study variables were pre- and post-treatment PPT, in addition to pre- and post-treatment measurements of the dorsal flexion of the ankle with the knee straight and flexed before and after treatment. ROM was measured in grades, and PPT was measured in Newtons.

Each variable had three recordings, and we used the mean of the three variables for statistical analyses.

### 2.6. Statistical Analysis

To check the normality of the data obtained we used the Shapiro–Wilk test as the study sample consisted of less than 30 subjects [36].

Data were considered normally distributed if *p* > 0.05. Descriptive statistical analysis was performed using the mean ± standard deviation (SD) and 95% confidence interval (CI). For the study of reliability, we examined the two types of reliability: (1) relative, which is the degree to which individuals maintain their position or value and (2) absolute, which is related to the degree of association with different measures of different individuals. We measured absolute reliability using the intraclass correlation coefficient (ICC) and absolute reliability with the standard error of the mean (SEM) as Bruton, Conway and Holgate and Landis and Koch [37] recommended in their studies and in other studies carried out for their reliability analysis [27,38,39].

Landis and Koch established a methodology to evaluate the result of the ICC value in relation to a mild reliability level, in which the ICC would be ≤0.20. A fair reliability will have a value between 0.21 and 0.40, a moderate reliability will have a reliability between 0.41 and 0.60, a re-liability will have a reliability between 0.61 and 0.80, and a reliability ≥ 0.81 will be considered almost perfect [37].

To assess the range of error for each dataset, the SEM value was calculated between two sessions from the ICC and SD using the formula: SEM = s_x.√(1 − r_xx) in which s_x was the standard deviation of the observed set of test scores, and r_xx was the reliability coefficient for these data which, in this case, was considered using the ICC.

Three tests of each variable were obtained for each situation (before and after), and the mean of three records was used to compare the before and after results. The Wilcoxon test was used to test for differences between parametric variables, and the paired t-test for parametric variables.

Based on the formula, VN = Mean +/−1.96* SD [40] derived from ultrasound and cadaveric dissection results, it was possible to define normality values (VN) using the result of each variable. The negative predictive value (NPV) was used to calculate the 95% CI. A *p*-value < 0.05 with a 95% CI was considered statistically significant for all tests (SPSS for Windows, version 26.0; SPSS Inc., Chicago, IL, USA).

The within-session reliability study of the variables and the normality values in the total population are shown in Table 2.

The minimum detectable change (MDC) was also calculated at a 95% confidence level, providing confidence that a change in the measurement was not the result of random variation or measurement error. This calculation was obtained from the SEM values using the formula: MDC = √2 × 1.96 × SEM. Both the SEM and the MDC were analysed according to Bland and Altman [40].

While a *p*-value can provide information as to whether or not a statistically significant difference between two groups exists, to determine how large this difference is, we need to calculate Cohen’s D, which is reflected in Table 3 and Table 4, and is interpreted as a value of 0.2 representing a small effect size, 0.5 representing a medium effect size, and 0.8 representing a large effect size [41].

## 3. Results

Table 1 shows the socio-demographic data of the study population based on sex. The reliability study can be seen in Table 2. It shows how the ICC reliability values were >0.95, which are considered by Landis and Koch to indicate perfect reliability. SEM and ICC values confirmed the reliability of the variables.

**Figure 4:**
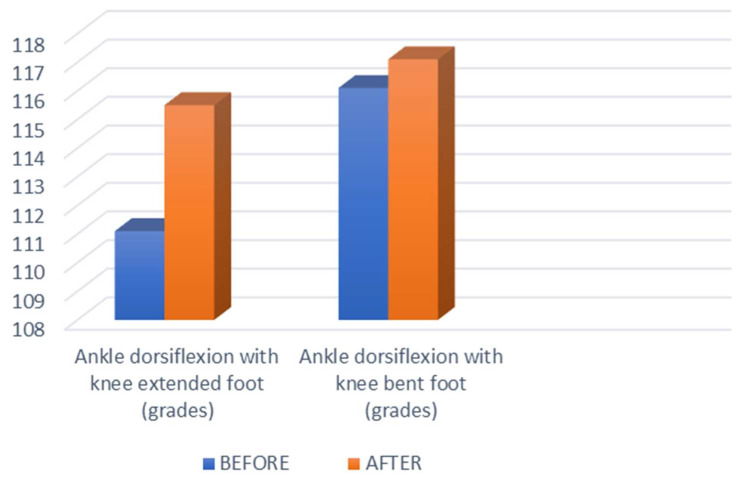
ROM of ankle dorsiflexion before and after Myofascial Induction Technique.

Figure 4 displays a representation of the comparative statistical study between ROM of ankle dorsiflexion before and after Myofascial Induction Technique, corresponding to the data shown in Table 3.

Table 3 shows the mean and standard deviation of the study variables before and after neural mobilization in addition to parametric or non-parametric comparison statistics to determine significant differences between the variables pre- and post-treatment. The variables that present a non-normal distribution (*p* < 0.05) are the foot last in the socio-demographic variables (Table 1) and the variable representing dorsal flexion of the ankle with flexed knee of the right ankle (Table 3).

Variable ankle dorsiflexion with knee bent shows a non-normal distribution (*p* < 0.05) in the ROM study. Ankle dorsiflexion with knee extended and PPT variables show a normal distribution as shown in Table 3 and Table 4.

**Figure 5:**
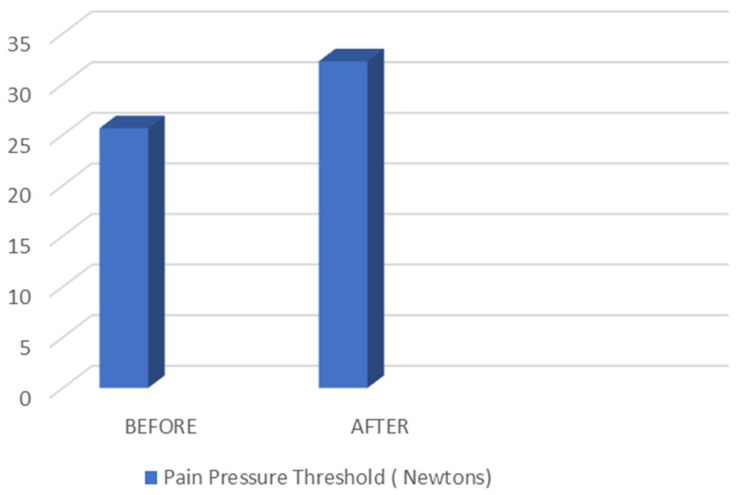
Pain Pressure Threshold before and after myofascial induction technique.

Figure 5 is the representation of the comparative statistical study between Pain Pressure Threshold before and after myofascial induction technique, corresponding to the data shown in Table 5.

Table 5 also shows Cohen’s D, where the effect size of the difference found can be observed.

## 4. Discussion

After evaluating the results of the present investigation, we found that the application of the superficial and deep technique proposed by Pilat does not improve ROM in terms of dorsal flexion of the ankle in the stretched or flexed knee position. The relationship of the PPT on the trigger point 1 of the gastrocnemius was found to produce an improvement in pain level on the trigger point after performing MIT.

The reliability analysis shows that the measurements of all variables are considered perfect reliability based on a study by Landis and Kock [37]. In fact, we obtained greater reliability than previous meta-analyses used to evaluate the weight-bearing lunge tests and obtained solid evidence that the inter-clinical reliability (ICC = 0.80–0.99) in addition to the intra-clinical reliability (ICC = 0.65–0.99) are good reliability tests. Furthermore, mean minimum detectable change scores of 4.6° for inter-clinical and 4.7° for intra-clinical were found in the latest meta-analysis [31] and when compared with our results, the minimum detectable change was 4.6° in this test. It may be necessary to emphasize that our results affirm that no increase in dorsiflexion due to above 4.6° occurred, and that for evaluating changes of lesser degree, further studies in other positions will be necessary. However, for ankle dorsiflexion over 4.6° we must therefore reject our hypothesis that MIT produces an improvement in ROM at latent gastrocnemius trigger points and retain the hypothesis that MIT produces a decrease in trigger point pain after MIT. A weight-bearing technique with an extended knee has proven to be reliable in our study, presenting a reliability greater than 0.90, considered by Landis and Koch to be almost perfect [37]. Different arithmetic means are observed before and after the technique without statistically significant differences in this population of healthy untrained individuals. Further studies are necessary to verify if there could be changes in other populations.

This study is the second study on the lower limb and the effects of the myofascial induction technique. The first study found that after application of the technique to the sole of the foot, the variables representing maximal forefoot pressure and forefoot surface area increased. In the opinion of the authors of this study, this increase could have been due to the reduction of fascial restrictions caused by the application of the technique [22]. On the other hand, application of the myofascial induction technique in chronic low back pain has been shown to increase joint flexion range [21]. We think that in non-trained healthy individuals the application of the myofascial induction technique in the calf does not produce an increase in dorsal ankle flexion of more than 4.6° or does not cause an increase at all because the fascial restrictions are less than in other active populations or those with muscular, neurological, or functional equine pathology.

Improving the ROM of ankles would be interesting from the point of view that its restriction causes functional ankle equinus condition [42,43], a condition associated with different pathologies [44], such as severe rupture of the anterior cruciate ligament, and increases in plantar pressure [42,43,44]. Although it is true that our subjects did not have dorsiflexion restrictions, future research in this population is necessary.

In relation to the other variable in our study, the PPT, only three studies that measure this variable after the use of the myofascial induction technique have been published. The first study did not find significant differences after application in the area of cervical myofascial induction on the C5–C6 zygapophyseal joint in healthy subjects [18]. The second study was carried out in healthy pain-free individuals and found no significant differences in maximal vertical mouth opening, in the PPT threshold mechanical sensitivity of the masticatory muscles, and in head posture [19]. Both studies demonstrate that in healthy subjects without pain, the MIT technique does not improve pressure pain. A third study was conducted with breast cancer survivors. A single myofascial induction session may partially improve mechanosensitive of median, radial, and ulnar nerves and yield positive effects on symptom mechanosensitive, especially regarding the ulnar nerve [45]. Due to these results, we consider that subjects who present with inflammation or pathology and painful symptoms, such as latent trigger points as in our study, will have an increase in their PPT after MIT application.

In this study, we verified that MIT is a global technique that when applied to the calf generates effects at trigger point 1 of the gastrocnemius, located close to the knee and not at the place of application of the technique, thus having distal effects. The mechanism by which trigger point pain decreases after its application should be the subject of further studies since it breaks the paradigm of many techniques that justify their effectiveness against myofascial syndrome by producing improvements in ROM, such as those associated with stretching. In addition, this study reveals the relationship between the trigger point and the fascia as a therapeutic unit and therefore implies that for myofascial pain syndrome, it is always necessary to treat the fascia in addition to the trigger point as other authors have reported. However, no studies until now have determined whether MIT could be used.

Based on current research, latent trigger points produce painful symptoms if stimulated, and painful mechanical stimulation is one such symptom. In fact, latent trigger points are more sensitive if they produce greater symptoms in response to the same stimulus. More sensitive trigger points produce greater symptoms and motor dysfunction, such as fatigue, decreased range of motion, development of cramps, decreased joint range, muscle weakness, deficits in fine muscle control, and alternating muscle activation patterns [2,46,47].

Therefore, most authors, including this research group, support the treatment of latent trigger points to prevent these motor dysfunctions and prevent the possibility of activating these trigger points in current research [2,9]. Therefore, we state that the umbral increase in mechanical stimulus threshold at which pain appears, measured in this research with the variable PPT, indicates the reduction in associated motor impairment and the possibility of activation of the trigger point. More investigations are certainly needed to ascertain the long-term effects regarding such an increase.

Due to publication of reviews and meta-analyses that indicate that MIT should not be used as a single therapy, future studies should determine the most appropriate protocol(s) to combine MIT and existing treatments, such as dry needling and conventional therapies. In relation to our results, we can affirm that MIT causes an increase in the pain threshold to pressure in the patent myofascial trigger points.

One of the limitations of this study is that to quantify the magnitude of this finding, it is necessary to conduct research on active trigger points since it may be that the technique has greater effects in cases where more significant pathology is present. Regarding joint range, it is also necessary to carry out further studies that can show whether this technique can have an effect in subjects with different pathologies in relation to increasing ankle joint range, while it may just produce no effect on healthy or untrained people. Another limitation is that this study does not discuss long-term effects, which we did evaluate, so future studies should study and compare the effects with other therapies in the long-term.

## 5. Conclusions

The results of this study demonstrate that MIT application to the calf improves the PPT at the latent MTP, but does not increase joint range in subjects without ankle dorsiflexion in healthy, non-trained without ROM restrictions.

## Figures and Tables

**Table 1:** Socio-demographic characteristics of the sample population.

Variable Total (*n* = 20 Calves)	Female Mean ± SD CI 95%	Male Mean ± SD CI 95%	Total Group Mean ± SD CI 95%	*p*
Age (years)	22.57 ± 4.89 (19.75–25.40)	23.33 ± 2.06 (21.17–25.50)	22.80 ± 4.20 (20.83–24.77)	0.153 ^a^
Body mass (Kg)	67 ± 9.36 (61.59–72.41)	58.67 ± 6.59 (51.75–65.59)	64.50 ± 9.31 (60.14–68.86)	0.051 ^a^
Height (cm)	170.71 ± 4.17 (168.30–173.13)	170.33 ± 1.36 (168.90–171.77)	170.60 ± 3.53 (168.95–172.25)	0.547 ^a^
BMI (Kg/m^2^)	22.93 ± 3.30 (21.02–24.84)	20.22 ± 2.29 (17.81–22.63)	22.11 ± 3.23 (20.60–23.63)	0.153 ^a^
Size of shoe	39.71 ± 1.72 (38.72–40.71)	41.00 ± 1.54 (39.37–42.63)	41.60 ± 7.56 (38.06–45.14)	0.444 ^a^

Abbreviation: Kg (Kilograms); cm (centimeters), BMI Body Mass Index), SD (standard deviation), CI 95% (confidence interval 95%). ^a^ non-normal distribution (U Mann–Whitney).

**Table 2:** Analysis of Intrasession Reliability of the ROM Variables Studied and Values of Normality in Total Population.

	Pre-Test (*n* = 20)			Post-Test (*n* = 20)		
Variable	ICC (95% CI)	SEM	Values of Normality 95% CI	ICC (95% CI)	SEM	Values of Normality 95% CI
Ankle dorsiflexion with knee extended foot (grades)	0.997 (0.993–0.999)	0.60	89.43–132.77	0.947 (0.889–0.978)	2.83	91.36–139.64
Ankle dorsiflexion with knee bent foot (grades)	0.961 (0.918–0.983)	1.38	101.37–128.83	0.989 (0.976–0.995)	1.79	83.65–150.55

Abbreviation: Kg (Kilograms); cm (centimeters), cm^2^ (centimeters^2^), SD (standard deviation), CI 95% (confidence interval 95%).

**Table 3:** ROM variables before and after Myofascial Induction Technique.

	Pre-Test (*n* = 20)		Post-Test (*n* = 20)			Effect Size
Variable	Mean ± SD (CI 95%)	Median (RI)	Mean ± SD (CI 95%)	Median (RI)	*p*	Cohen’s D
Ankle dorsiflexion with knee extended foot (grades)	111.1 ± 11.06 (105.92–116.27)	113.83 (16.16)	115.50 ± 12.32 (109.73–121.26)	118.00 (12.32)	0.069 ^b,^*	−0.431
Ankle dorsiflexion with knee bent foot (grades)	115.10 ± 7.01 (112.81–119.38)	117.83 (7.91)	117.10 ± 17.07 (113.17–121.02)	116.00 (10.08)	0.420 ^a,^*	−0.260

Abbreviations: SD, Standard Deviation; CI 95%, Confidence interval 95%; RI, Range interquartile; grades; ^a^ *p* value in from Wilcoxon Signed-Rank Test; ^b^ *p* value from paired *t*-test; A *p* value < 0.05 with a confidence interval of 95% was considered statistically significant, * statistical significance.

**Table 4:** Analysis of Intrasession Reliability of the Pain Pressure Threshold variables.

	Pre-Test (*n* = 20)			Post-Test (*n* = 20)		
Variable	ICC (95% CI)	SEM	Values of Normality 95% CI	ICC (95% CI)	SEM	Values of Normality 95% CI
Pain Pressure Threshold gastrocnemius (N)	0.979 (0.957–0.991)	1.65	3.28–48.03	0.927 (0.847–0.969)	3.87	4.17–60.33

Abbreviation: Kg (Kilograms); cm (centimeters), cm^2^ (centimeters^2^), SD (standard deviation), CI 95% (confidence interval 95%).

**Table 5:** Pain Pressure Threshold variables before and after Myofascial Induction Technique.

	Pre-Test (*n* = 20)		Post-Test (*n* = 20)			Effect Size
Variable	Mean ± SD (CI 95%)	Median (RI)	Mean ± SD (CI 95%)	Median (RI)	*p*	Cohen’s D
Pain Pressure Threshold gastrocnemius (N)	25.64 ± 11.41 (20.29–30.98)	22.75 (13.81)	32.25 ± 14.33 (25.53–38.96)	29.36 (17.22)	0.001 ^b,^*	−0.855

Abbreviations: SD, Standard Deviation; CI 95%, Confidence interval 95%; RI, Range interquartile; N, Newtons; ^b^ *p* value from paired *t*-test; A *p* value < 0.05 with a confidence interval of 95% was considered statistically significant, * statistical significance.

## Data Availability

The data are available upon request from the corresponding author.
